# Supplementary material for: TDP1 suppresses chromosomal translocations and cell death induced by abortive TOP1 activity during gene transcription
Source: Nat Commun. 2023 Nov 9;14:6940. doi: 10.1038/s41467-023-42622-7 (PMC10636166; doi:10.1038/s41467-023-42622-7)
Supplement: Supplementary file 1 — Supplementary Information [file 41467_2023_42622_MOESM1_ESM.pdf]

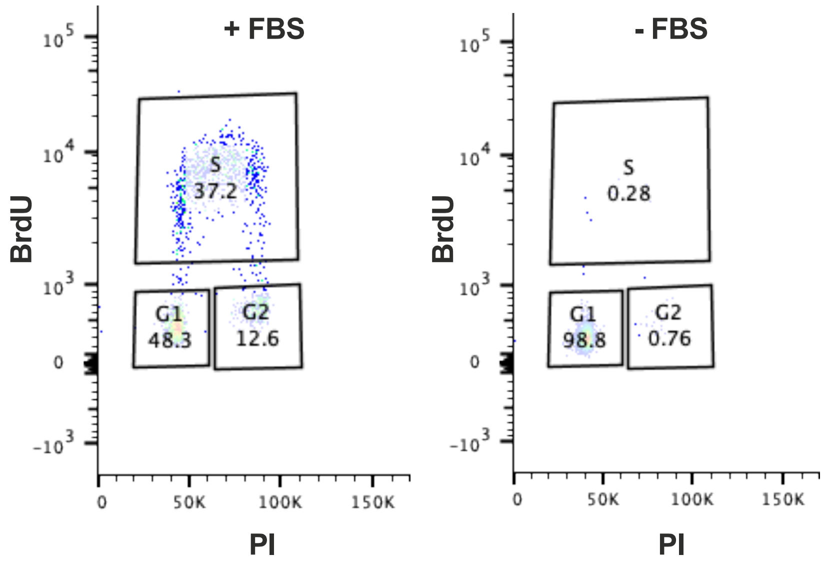

Figure S1. Serum-starved confluent RPE-1 cells synchronised in G0/G1. RPE-1 cells were stained for incorporated BrdU against total DNA content using propidium iodide (PI). Left, proliferating cells in presence of serum (+ FBS). Right, serum-starved confluent cells (- FBS). The percentage of each phase is indicated.

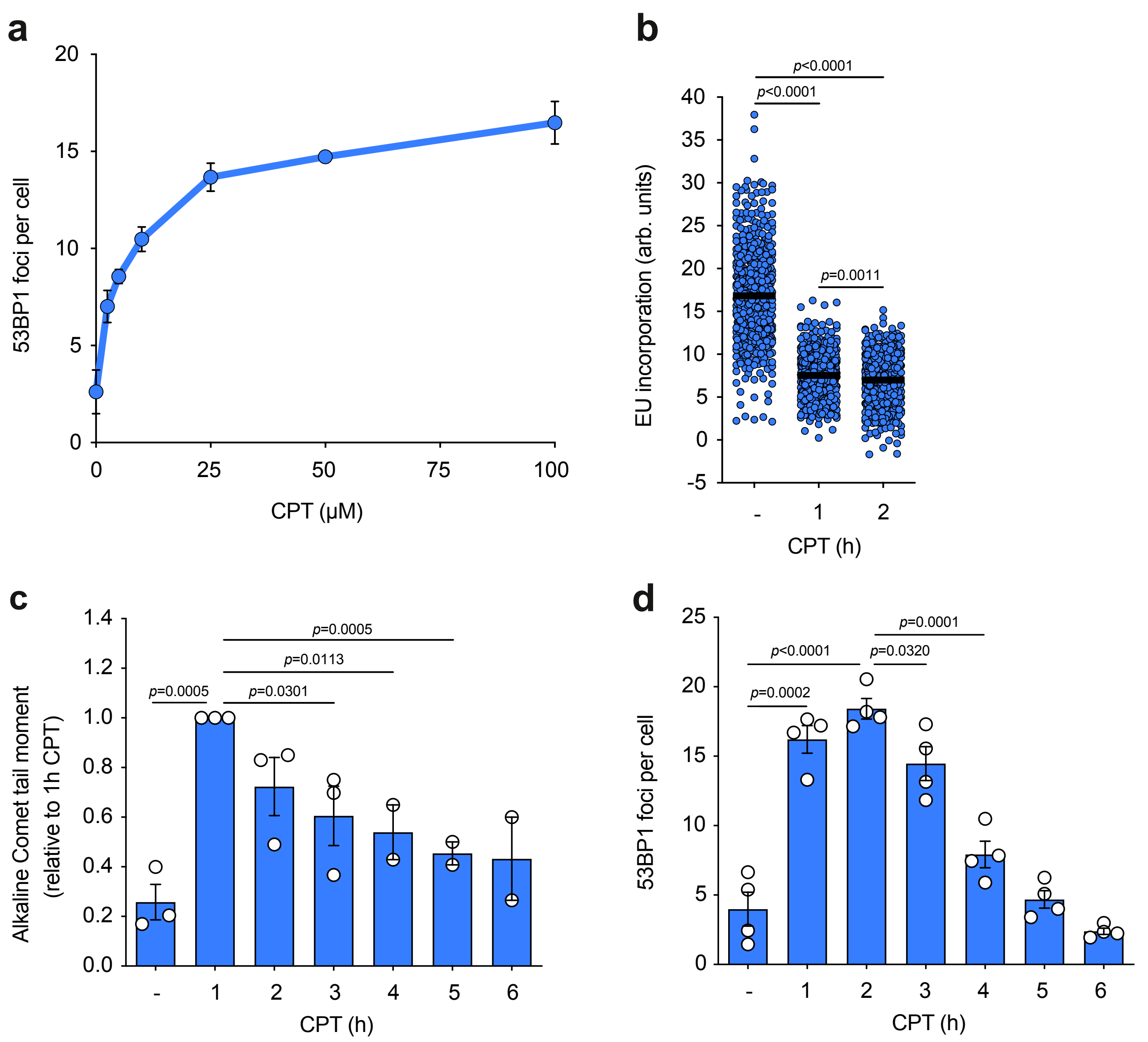

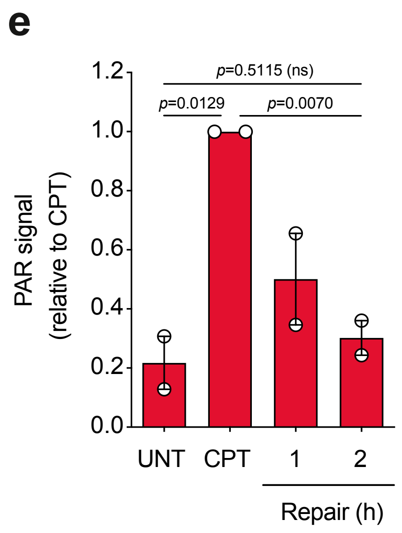

Figure S2. DNA damage and transcription inhibition G0/G1 RPE-1 cells upon CPT treatment.

a 53BP1 foci in serum-starved RPE-1 cells treated with CPT for 1 h. *n* = 3 independent experiments. **b** Quantification of EU signal in serum-starved RPE-1 cells treated with CPT (25 μM) for the indicated hours. From left to right: *n* = 549, *n* = 604 and *n* = 535 cells over two independent experiments. **c** Detection of DNA breaks by alkaline comet assay in serum-starved RPE-1 cells treated with CPT (25 μM) for the indicated hours. *n* ≥ 2 independent experiments. **d** 53BP1 foci in serum-starved RPE-1 cells treated with CPT (25 μM) for the indicated hours. *n* = 4 independent experiments. **e** Quantification of PAR by immunofluorescence in serum-starved *TDP1*$^{-/-}$ RPE-1 cells treated with CPT (25 μM) for 2 h, and after the indicated repair periods in drug-free medium. *n* = 2 independent experiments. UNT untreated. Data were represented as mean ±SEM. Statistical significance was determined by two-tailed unpaired *t*-test for **b**-**e**. ns non-significance. Source data are provided as a Source Data file.

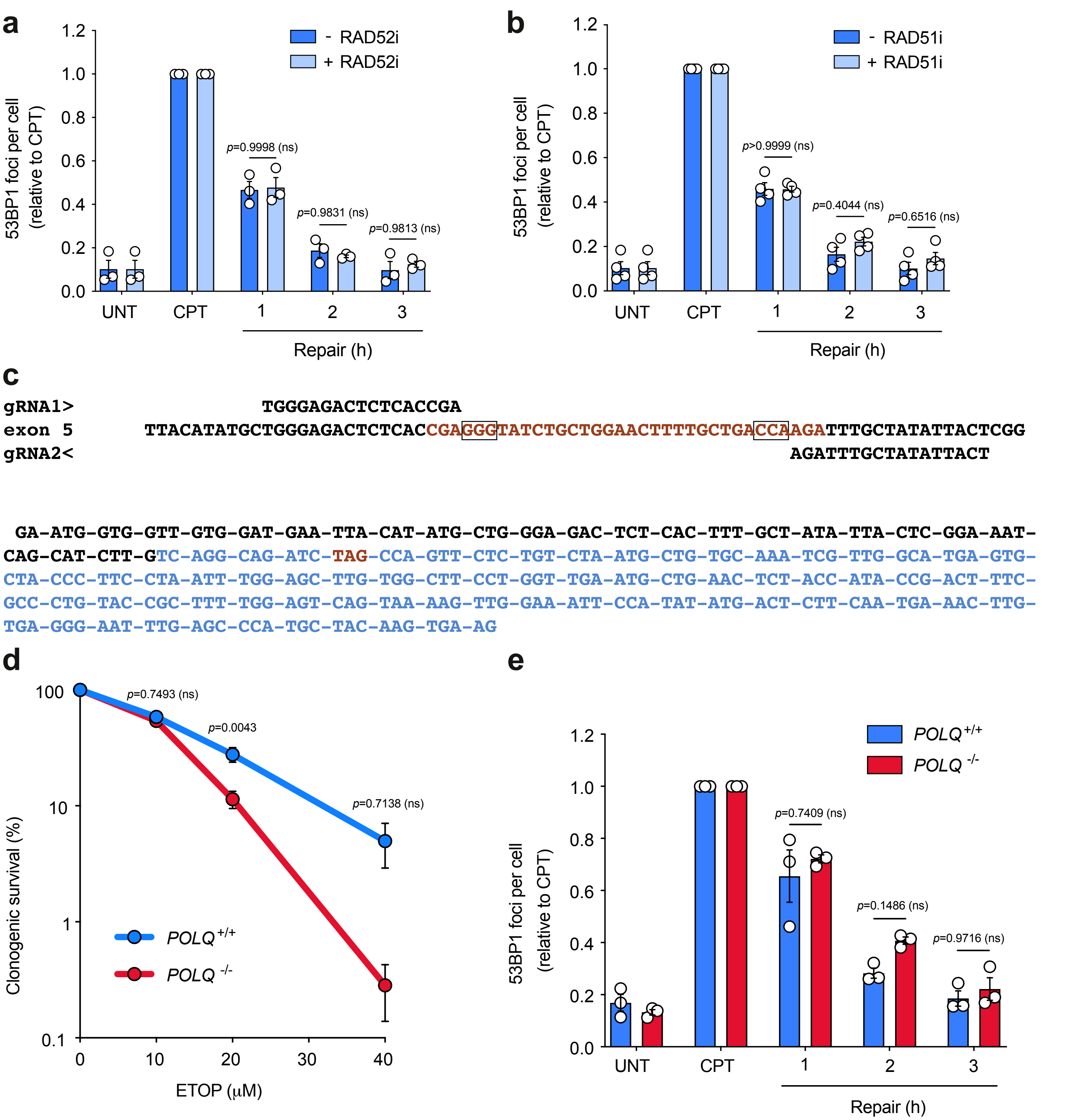

Figure S3. Alternative pathways to cNHEJ-dependent TOP1-induced DSB repair.

a-b 53BP1 foci in serum-starved RPE-1 cells after 1 h treatment with 25 µM CPT, and during repair in drug-free medium. Where indicated, cells were incubated with the RAD52 inhibitor AICAR (40 µM) (**a**) or with the RAD51 inhibitor RI-1 (20 µM) (**b**) during repair. $n = 3$ (**a**) and $n = 4$ (**b**) independent experiments. **c** Genomic sequence of *POLQ* exon 5 and CRISPR sgRNAs 1 and 2 employed for *POLQ* mutagenesis. Frames indicate PAM sequences. Clones were selected by PCR and sequenced. Bases in red correspond to a 34-nucleotide deletion found in homocygosis in all selected clones (top). Deletion mutation in *POLQ*. Exons 5 (black) and 6 (blue) are shown. The 34 bp deletion in exon 5 generates a premature stop codon in exon 6 (indicated in red) (bottom). **d** Clonogenic survival of *POLQ*$^{+/+}$ and *POLQ*$^{-/-}$ RPE-1 cells treated with etoposide (ETOP) for 3 h. $n = 3$ independent experiments. **e** 53BP1 foci in serum-starved *POLQ*$^{+/+}$ and *POLQ*$^{-/-}$ cells after 1 h treatment with 25 µM CPT, and during repair in drug-free medium. $n = 3$ independent experiments. UNT untreated. Data were represented as mean ±SEM. Statistical significance was determined by two-way ANOVA followed by Sidak's multiple comparisons test for **a**, **b**, **d** and **e**. ns non-significance. Source data are provided as a Source Data file.

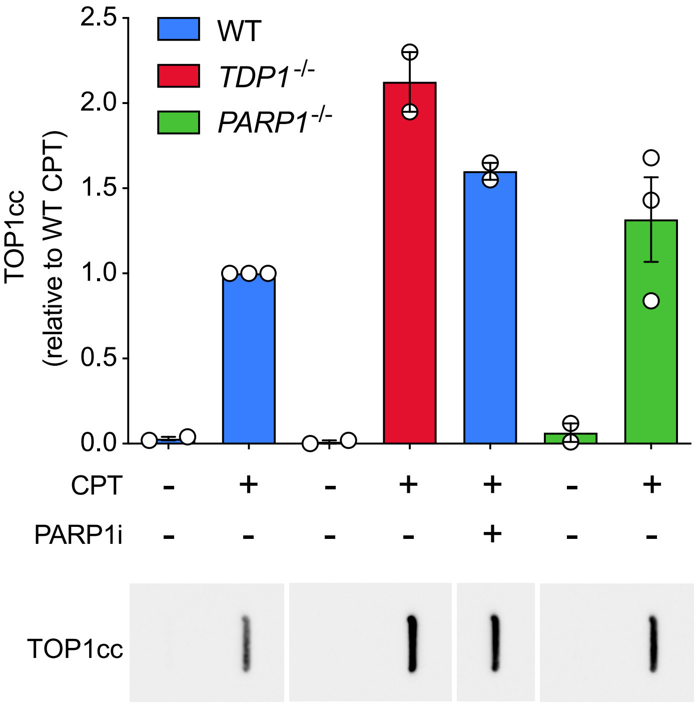

Figure S4. Analysis of TOP1 cleavage-complexes (TOP1cc) by ICE assay.
Serum-starved wild-type (WT), *PARP1*$^{-/-}$ and *TDP1*$^{-/-}$ RPE-1 cells were treated with 25 µM CPT for 1 h. Where indicated, cells were pre-incubated with the PARP inhibitor KU58948 (1 µM) for 1 h prior to and during CPT treatment. $n \geq 2$ independent experiments. Data were represented as mean ±SEM. Representative plots of TOP1cc from same replicate are shown. Source data are provided as a Source Data file.

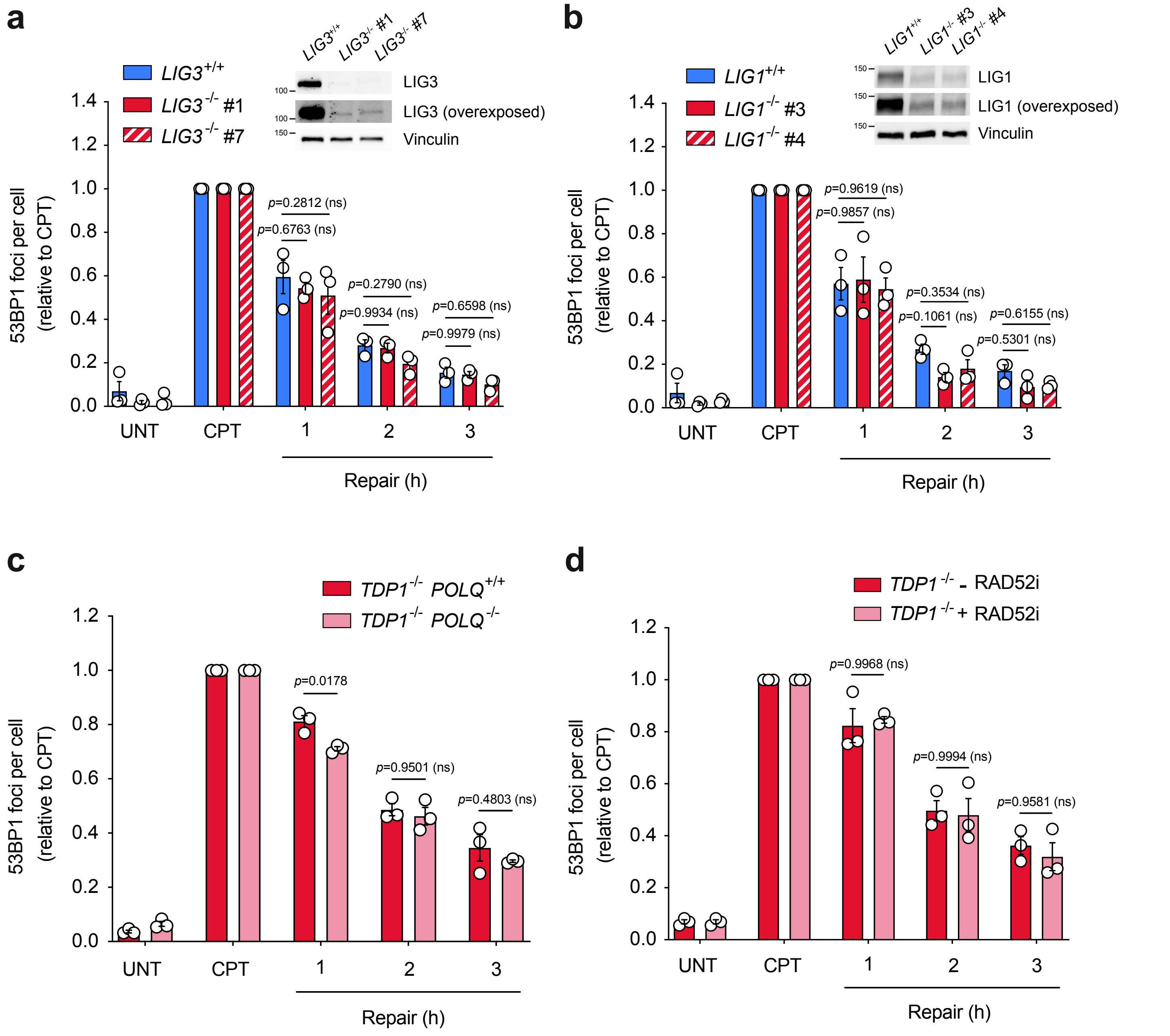

Figure S5. DNA repair ligases in TOP1-induced DSB repair.
a-b 53BP1 foci in serum-starved *LIG3*$^{+/+}$ and two *LIG3*$^{-/-}$ RPE-1 clones (**a**) and in serum-starved *LIG1*$^{+/+}$ and two *LIG1*$^{-/-}$ RPE-1 clones (**b**) after 1 h treatment with 12.5 µM CPT, and during repair in drug-free medium. *n* = 3 independent experiments. Protein blot of LIG3 and LIG1 are shown. Vinculin was used as a loading control. Molecular weight markers are in KDa. **c** 53BP1 foci in serum-starved *TDP1*$^{-/-}$ *POLQ*$^{+/+}$ and *TDP1*$^{-/-}$ *POLQ*$^{-/-}$ RPE-1 cells after 1 h treatment with 12.5 µM CPT, and during repair in drug-free medium. *n* = 3 independent experiments. **d** 53BP1 foci in serum-starved *TDP1*$^{-/-}$ cells after 1 h treatment with 12.5 µM CPT, and during repair in drug-free medium. Where indicated, cells were incubated with the RAD52 inhibitor AICAR (40 µM) during repair. *n* = 3 independent experiments. UNT untreated. Data were represented as mean ±SEM. Statistical significance was determined by two-way ANOVA followed by Sidak's multiple comparisons test for **a-d**. ns non-significance. Source data are provided as a Source Data file.

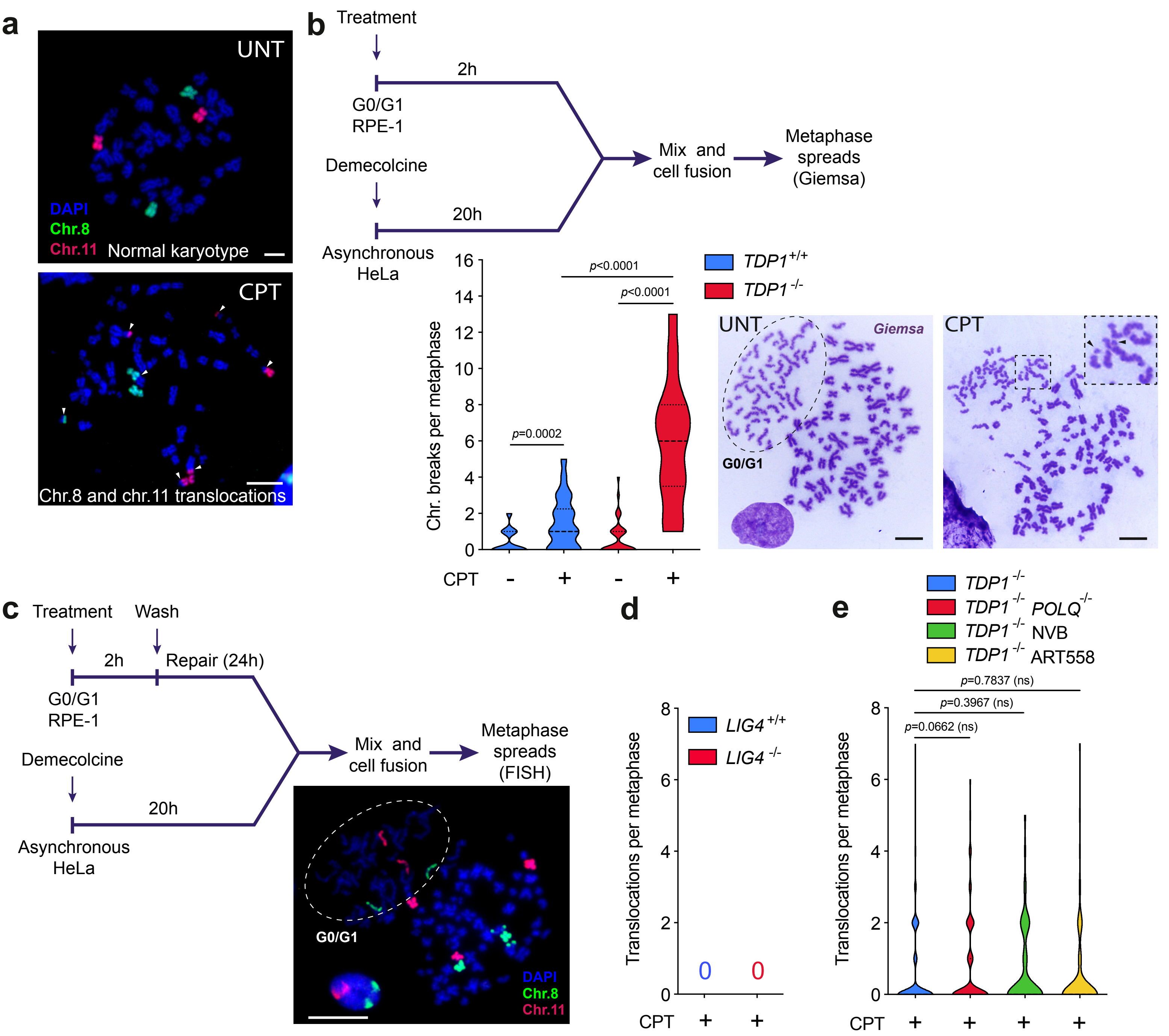

Figure S6. Workflow to study chromosomal translocations in G0/G1 cells.

a FISH images of RPE-1 cells in metaphase with normal karyotype vs. translocation of chromosomes 8 (green) and 11 (red) upon CPT treatment followed by repair. DAPI counterstain (blue) is shown. White arrows indicate translocation events. **b** Top, schematic of the premature chromosome condensation (PCC) in G0/G1 cells workflow. Bottom, frequency of chromosomal breaks was quantified in *TDP1*$^{+/+}$ and *TDP1*$^{-/-}$ RPE-1 cells after CPT treatment (25 µM) for 2 h. From left to right: *n* = 35, *n* = 42, *n* = 34 and *n* = 33 cells over two independent experiments. Inset, Giemsa images of G0/G1 RPE-1 cells (dotted ellipse) fused with HeLa cells synchronized in metaphase. Black arrows indicate break events. **c** Top, schematic of the PCC in G0/G1 cells workflow. Bottom, FISH image of G0/G1 RPE-1 cells (dotted ellipse) fused with HeLa cells synchronized in metaphase. Chromosomes 8 (green) and 11 (red), and DAPI counterstain (blue) are shown. **d**-**e** Translocation frequencies in serum-starved *LIG4*$^{+/+}$, *LIG4*$^{-/-}$, *TDP1*$^{-/-}$ and *TDP1*$^{-/-}$ *POLQ*$^{-/-}$ RPE-1 cells in metaphase spreads prepared 48 h after CPT treatment (25 µM) for 2 h followed by 6 h repair in drug-free medium. Where indicated, cells were pre-treated with novobiocin (NVB) (100 µM) or ART558 (10 µM) for 30 min prior to, during, and 6 h after CPT treatment. From left to right: *n* = 50 and *n* = 50 cells over two independent experiments for **d** and *n* = 300, *n* = 161, *n* = 53 and *n* = 83 cells over at least two independent experiments for **e**. UNT untreated. Data were represented as mean ±SEM. Statistical significance was determined by two-tailed unpaired *t*-test for **b** and **e**. Scale bar, 10 µm for **a**-**c**. ns non-significance. Source data are provided as a Source Data file.

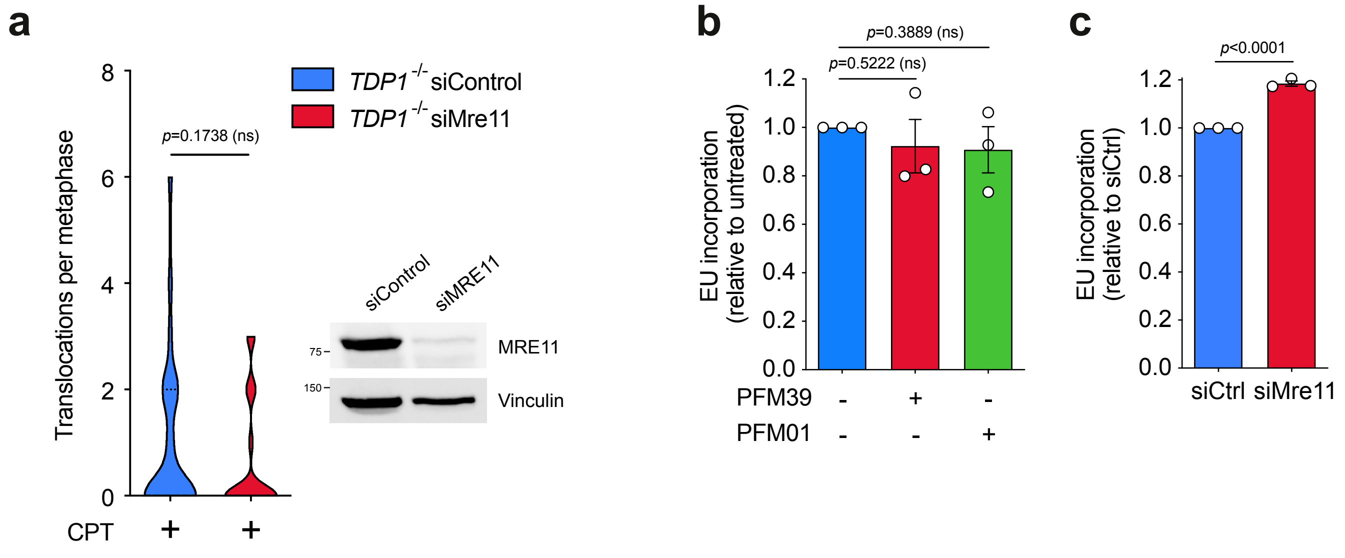

Figure S7. siMRE11 and PFM01 do not inhibit transcription.
a Translocation frequencies in serum-starved mock-depleted (siControl) or MRE11-depleted (siMRE11) *TDP1*$^{-/-}$ RPE-1 cells in metaphase spreads prepared 48 h after CPT treatment (25 µM) for 2 h followed by 6 h repair in drug-free medium. From left to right: *n* = 54 and *n* = 39 cells over two independent experiments. Protein blot of MRE11 is shown. Vinculin was used as a loading control. Molecular weight markers are in kDa. **b** Quantification of EU signal in serum-starved RPE-1 cells treated with PFM39 (25 µM) or PFM01 (10 µM) for 1 h. *n* = 3 independent experiments. **c** Quantification of EU signal in serum-starved mock-depleted (siCtrl) or MRE11-depleted (siMRE11) RPE-1 cells. Other details as in **b**. Data were represented as mean ±SEM. Statistical significance was determined by two-tailed unpaired *t*-test for **a**-**c**. ns non-significance. Source data are provided as a Source Data file.

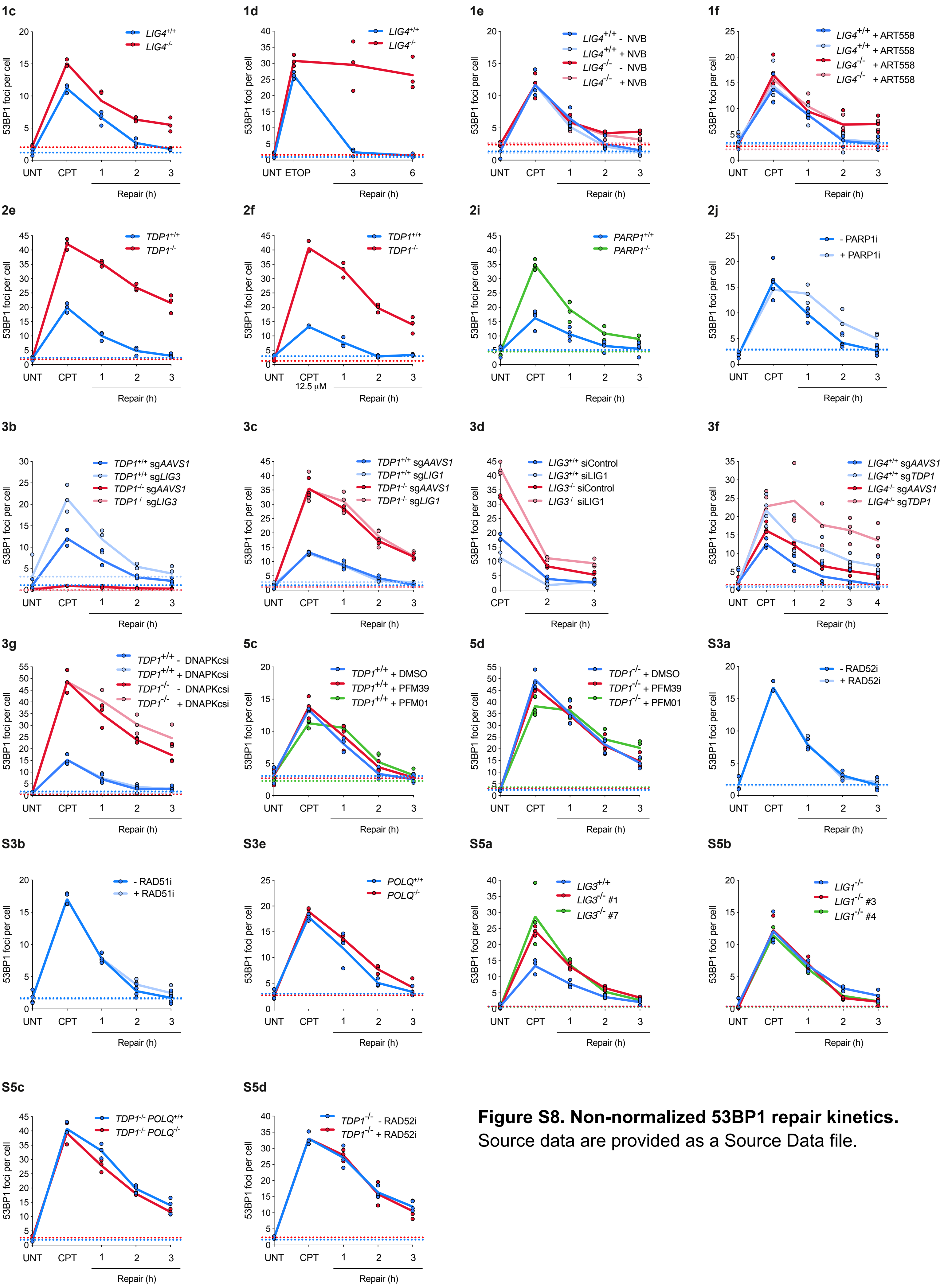

Figure S8. Non-normalized 53BP1 repair kinetics.
Source data are provided as a Source Data file.

Table S1. Target sequences used in this study for sgRNAs.

Target Gene	Target sequence
POLQ #1	TGGGAGACTCTCACCGA
POLQ #2	AGTAATATAGCAAATCT
LIG3 #1	GGCCACCACAAAAAAAATCG
LIG3 #2	CTTGGCTGACATGATAACCC
AAVS1	GGGGCCACTAGGGACAGGAT
TDP1	TCTTTGGGCAGTGCCGTCAT
LIG1	GAGAGGGAAGCATTGTTCTC
