# Supplementary material for: TDP1 suppresses chromosomal translocations and cell death induced by abortive TOP1 activity during gene transcription
Source: Nat Commun. 2023 Nov 9;14:6940. doi: 10.1038/s41467-023-42622-7 (PMC10636166; doi:10.1038/s41467-023-42622-7)
Supplement: Supplementary file 3 — Reporting Summary [file 41467_2023_42622_MOESM3_ESM.pdf]

nature portfolio

Corresponding author(s): Fernando Gómez-Herreros

Last updated by author(s): Oct 5, 2023

Reporting Summary

Nature Portfolio wishes to improve the reproducibility of the work that we publish. This form provides structure for consistency and transparency in reporting. For further information on Nature Portfolio policies, see our Editorial Policies and the Editorial Policy Checklist.

Statistics

For all statistical analyses, confirm that the following items are present in the figure legend, table legend, main text, or Methods section.

n/a	Confirmed	
☐	☒	The exact sample size (*n*) for each experimental group/condition, given as a discrete number and unit of measurement
☐	☒	A statement on whether measurements were taken from distinct samples or whether the same sample was measured repeatedly
☐	☒	The statistical test(s) used AND whether they are one- or two-sided<br>*Only common tests should be described solely by name; describe more complex techniques in the Methods section.*
☒	☐	A description of all covariates tested
☐	☒	A description of any assumptions or corrections, such as tests of normality and adjustment for multiple comparisons
☐	☒	A full description of the statistical parameters including central tendency (e.g. means) or other basic estimates (e.g. regression coefficient) AND variation (e.g. standard deviation) or associated estimates of uncertainty (e.g. confidence intervals)
☐	☒	For null hypothesis testing, the test statistic (e.g. *F*, *t*, *r*) with confidence intervals, effect sizes, degrees of freedom and *P* value noted<br>*Give P values as exact values whenever suitable.*
☒	☐	For Bayesian analysis, information on the choice of priors and Markov chain Monte Carlo settings
☒	☐	For hierarchical and complex designs, identification of the appropriate level for tests and full reporting of outcomes
☒	☐	Estimates of effect sizes (e.g. Cohen's *d*, Pearson's *r*), indicating how they were calculated

Our web collection on statistics for biologists contains articles on many of the points above.

Software and code

Policy information about availability of computer code

Data collection	Microscopy: Olympus BX-61<br>Immunoblots: BioRad Chemidoc Touch<br>FACS: BD FACSCanto II flow cytometer
Data analysis	CometScore Pro Software, comet analysis<br>Image Lab 6.0.0, chemiluminescence analysis<br>Image J 1.52d, image analysis<br>BD FACSDiva Software v9.0., FACS analysis<br>GraphPad Prism 8.2.1, numerical and statistical analysis<br>Adobe Illustrator 24.0, figure assembly

For manuscripts utilizing custom algorithms or software that are central to the research but not yet described in published literature, software must be made available to editors and reviewers. We strongly encourage code deposition in a community repository (e.g. GitHub). See the Nature Portfolio guidelines for submitting code & software for further information.

Data

Policy information about availability of data

All manuscripts must include a data availability statement. This statement should provide the following information, where applicable:

- Accession codes, unique identifiers, or web links for publicly available datasets
- A description of any restrictions on data availability
- For clinical datasets or third party data, please ensure that the statement adheres to our policy

Source data are provided with this paper. Uncropped blots including 1C, 2A, 2H, 3A, 3B, 3C, 3D, 3E, 5B, S4, S5A, S5B and S7A are provided with this paper.

Human research participants

Policy information about studies involving human research participants and Sex and Gender in Research.

Reporting on sex and gender	These studies have not involved human research participants
Population characteristics	These studies have not involved human research participants
Recruitment	These studies have not involved human research participants
Ethics oversight	These studies have not involved human research participants

Note that full information on the approval of the study protocol must also be provided in the manuscript.

Field-specific reporting

Please select the one below that is the best fit for your research. If you are not sure, read the appropriate sections before making your selection.

☒ Life sciences ☐ Behavioural & social sciences ☐ Ecological, evolutionary & environmental sciences

For a reference copy of the document with all sections, see nature.com/documents/nr-reporting-summary-flat.pdf

Life sciences study design

All studies must disclose on these points even when the disclosure is negative.

Sample size	No statistical method was used to predetermine sample size. Unless indicated, at least three independent biological replicates was considered as the minimal/sufficient number of replicates based on previous publications.
Data exclusions	No exclusion was applied.
Replication	For all experiments, the number of biological replicates is indicated and reproduced the representative data are shown in figures.
Randomization	No human participants or animal models were reported in this manuscript. Samples were organized into different treatments (cell type/ chemical treatment/knockdown or mutant conditions).
Blinding	Investigators were blinded during clonogenic assays, foci, chromosomal breaks and translocation scoring. For the automated scoring of comet tail moment blinding was not necessary due to intrinsically unbiased nature of the approach. For the quantification of chemiluminescence (ICE) and immunofluorescence (nascent RNA by EU, poly ADP-ribosylation) blinding was not necessary due to intrinsically unbiased nature of the approach.

Reporting for specific materials, systems and methods

We require information from authors about some types of materials, experimental systems and methods used in many studies. Here, indicate whether each material, system or method listed is relevant to your study. If you are not sure if a list item applies to your research, read the appropriate section before selecting a response.

Materials & experimental systems

n/a	Involved in the study
☐	☒ Antibodies
☐	☒ Eukaryotic cell lines
☒	☐ Palaeontology and archaeology
☒	☐ Animals and other organisms
☒	☐ Clinical data
☒	☐ Dual use research of concern

Methods

n/a	Involved in the study
☒	☐ ChIP-seq
☐	☒ Flow cytometry
☒	☐ MRI-based neuroimaging

Antibodies

Antibodies used

Primary antibodies:
LIG1 (Santa Cruz B, sc-47703) 1:1000
LIG3 (GeneTex, GTX70143) 1:1000
LIG4 (Santa Cruz B, sc-271299) 1:100
MRE11 (Novus Biologicals, NB100-142) 1:5000
gammaH2AX (Millipore, 05-636) 1:1000
TDP1 (Santa Cruz B, sc-365674) 1:250
TOP1cc (Millipore, MABE1084) 1:250
Vinculin (Santa Cruz B, sc-25336) 1:1000
V5-tag (Abcam, ab15828) 1:2000.
anti-53BP1 (Novus Biologicals, NB100-904) 1:2500
anti-BrdU (SantaCRuz, sc-32323) 1:1000
PARP1 (ThermoFisher Scientific, 436400) 1:1000

Secondary antibodies:
Chemiluminescence (1:5000 dilution in Tris buffered saline buffer, 0.1% Tween20, 1% BSA): HRP-bovine anti-goat IgG (H+L), HRP-goat anti-mouse IgG (H+L) and HRP-goat anti-rabbit IgG (H+L) (Jackson ImmunoResearch 805-035-180, 115-035-146 and 115-035-144 respectively).
Immunofluorescence (1:1000 dilution in phosphate buffered saline buffer, 1% BSA): Alexa Fluor 488-goat anti-mouse IgG (H+L), Alexa Fluor 488-goat anti-rabbit IgG (H+L), Alexa Fluor 546-goat anti-mouse IgG (H+L), Alexa Fluor 546-goat anti-rabbit IgG (H+L) (ThermoFisher Scientific A11001, A11008, A11003 and A11010 respectively).

Validation

Methods of validation and references to published application for all primary antibodies are all present into manufacturer dedicated website page of each indicated product.
DNA Ligase I Antibody (1A9) (Santa Cruz B, sc-47703) is a mouse monoclonal IgG1κ antibody. Reactivity: mouse, rat, bovine and human. Applications: WB, IP and ELISA. It has been validated by WB in Jurkat whole cell lysate and in MOLT-4, PC-3 and F9 whole cell lysates, and cited in 4 publications according to manufacturer’s website (https://www.scbt.com/es/p/dna-ligase-i-antibody-1a9).
DNA Ligase III Antibody (1F3) (GeneTex, GTX70143) is a mouse monoclonal IgG1 antibody. Reactivity: human, mouse, chicken. Applications: WB, ICC/IF, IP, Blocking, PLA. It has been validated by WB in 293T whole cell extracts, and cited in 24 publications according to manufacturer’s website (https://www.genetex.com/Product/Detail/DNA-ligase-III-antibody-1F3/GTX70143).
DNA Ligase IV Antibody (D-8) (Santa Cruz B, sc-271299) is a mouse monoclonal IgG2aκ antibody. Reactivity: mouse, rat and human. Applications: WB, IP, IF and ELISA. It has been validated by WB in HeLa, JAR, KNRK, Jurkat, Ramos and AN3 CA whole cell lysates, rat thymus tissue extract, and in HeLa and Ramos nuclear extracts, and cited in 25 publications according to manufacturer’s website (https://www.scbt.com/es/p/dna-ligase-iv-antibody-d-8).
MRE11 (Novus Biologicals, NB100-142) is a rabbit polyclonal IgG antibody. Reactivity: human, mouse, rat, chicken, hamster, bovine canine, primate, equine and feline. Applications: WB, Simple Western, ELISA, Flow, Func, ICC/IF, IHC, IP, WB, ChIP, KD, KO. It has been validated by WB in HeLa lysate, by ICC and IF in HeLa cells, by IHC staining in human epidermis, by WB in HeLa and MEF lysates, and cited in 218 publications according to manufacturer’s website (https://www.novusbio.com/products/mre11-antibody_nb100-142).
Anti-phospho-Histone H2A.X (Ser139) (Millipore, 05-636), clone JBW301 is a mouse monoclonal IgG1 antibody. Reactivity: Vertebrates. Applications: ICC, IF, ChIP, IHC. It has been validated by ChIP, ICC, IF and WB (i.e., validated by ICC in Jurkat cells and by WB in Jurkat cells lysates), and cited in 496 publications according to manufacturer’s website (https://www.merckmillipore.com/ES/es/product/Anti-phospho-Histone-H2A.X-Ser139-Antibody-clone-JBW301,MM_NF-05-636).
TDP1 Antibody (C-3) (Santa Cruz B, sc-365674) is a mouse monoclonal IgG1κ antibody. Reactivity: human. Applications: WB, IP, IF, IHC(P) and ELISA. It has been validated by WB and infrared WB in Ramos whole cell lysate, by immunoperoxidase staining of formalin in fixed, paraffin-embedded human thyroid gland tissue, and cited in 9 publications according to manufacturer’s website (https://www.scbt.com/p/tdp1-antibody-c-3).
Anti-Topoisomerase I-DNA Covalent Complexes Antibody, clone 1.1A (Millipore, MABE1084) is a mouse monoclonal IgG2bκ antibody. Reactivity: human, mouse. Applications: DB, ELISA, FC, ICC. It has been validated by ICC and IF in A549 human lung carcinoma cells, by FC in HCT116 human colon cancer cells, and cited in 11 publications according to manufacturer’s website (https://www.merckmillipore.com/ES/es/product/Anti-Topoisomerase-I-DNA-Covalent-Complexes-Antibody-clone-1.1A,MM_NF-MABE1084?ReferrerURL=https%3A%2F%2Fwww.google.com%2F).
Vinculin Antibody (H-10) (Santa Cruz B, sc-25336) is a mouse monoclonal IgG2aκ antibody. Reactivity: human, mouse and rat. Applications: WB, IP, IF, IHC(P) and ELISA. It has been validated by direct fluorescent WB analysis in Sol8, A-10, K-562 , PC-3, HEL 92.1.7 and BC3H1 whole cell lysates, by WB in HeLa, 293T, HUV,EC-E, HISM, U-937, SJRH30, MDCK, K-562, PC-3, Sol8, BC3H1, HEL 92.1.7 and A-10 whole cell lysates and rat heart tissue extract, by immunoperoxidase staining of formalin in fixed, paraffin-embedded human placenta, human bone marrow, human testis and human breast tissues, by IF staining of methanol-fixed HeLa cells, and cited in 166 publications according to manufacturer’s website (https://www.scbt.com/es/p/vinculin-antibody-h-10).
Anti-V5 tag antibody (Abcam, ab15828) 1:2000 is a rabbit polyclonal IgG antibody. Reactivity: species independent. Applications: ChIP, WB. It has been validated by WB and ChIP in 293T human cell line, and cited in 49 publications according to manufacturer’s

website (https://www.abcam.com/products/primary-antibodies/v5-tag-antibody-ab15828.html).
53BP1 Antibody - BSA Free (Novus Biologicals, NB100-904) is a rabbit polyclonal IgG antibody. Reactivity: human, mouse, primate, and primate-orangutan. Applications: WB, Flow, Func, ICC/IF, IHC, IP, KD. It has been validated by WB in A2780 cells, U2OS cells, by ICC/IF in NIH3T3 cells, HeLa cells, C33A cells, human renal cancer cells, LNCaP cells, Ntera2 cells, by FC in HeLa and Ntera2 cells, and cited in 112 publications according to manufacturer's website (https://www.novusbio.com/products/53bp1-antibody_nb100-904).
BrdU Antibody (IIB5) (SantaCRuz, sc-32323) is a mouse monoclonal IgG1κ antibody. Applications: IP, IHC(P), ELISA, IF, FCM. It has been validated by IF in HeLa cells and by FC in K-562 cells, and cited in 241 publications according to manufacturer's website (https://www.scbt.com/es/p/brdu-antibody-iib5).
PARP1 Monoclonal Antibody (123), (Invitrogen, 436400) is a mouse monoclonal IgG1 antibody. Reactivity: Canine, Equine, Human, Mouse, Rat, Rhesus Monkey. Applications: ICC, IF, IHC(P), IP, WB. It has been validated by WB in Jurkat, HeLa and MDA-MB-231 cells, by ICC/IF in A549 cells, by IHC(P) in human diffuse large B cell lymphoma tissue, and cited in 11 publications according to manufacturer's website (https://www.fishersci.es/shop/products/parp1-monoclonal-antibody-123-invitrogen/10257464).

Eukaryotic cell lines

Policy information about cell lines and Sex and Gender in Research

Cell line source(s)	hTERT RPE-1 cells (CRL-4000, ATCC)<br>HeLa cells (CRL-CCL-2, ATCC)
Authentication	Cell lines were purchased from ATCC (authenticated by STR profiling).
Mycoplasma contamination	All cell lines were regularly tested for mycoplasma contamination (MycoAlert ® Mycoplasma Detection Kit LONZA LT07). In all cases cells tested negative for mycoplasma contamination.
Commonly misidentified lines<br>(See ICLAC register)	No commonly misidentified cell lines were used in this study.

Flow Cytometry

Plots

Confirm that:

☒ The axis labels state the marker and fluorochrome used (e.g. CD4-FITC).

☒ The axis scales are clearly visible. Include numbers along axes only for bottom left plot of group (a 'group' is an analysis of identical markers).

☒ All plots are contour plots with outliers or pseudocolor plots.

☒ A numerical value for number of cells or percentage (with statistics) is provided.

Methodology

Sample preparation	Sample preparation is indicated in Methods
Instrument	Canto II (Becton Dickinson)
Software	FlowJo (Becton Dickinson)
Cell population abundance	No sorting method was used
Gating strategy	Cells were gated from plotting FSC-A/SSC-A. Doublet exclusion was performed by plotting the height or width against the area for forward scatter or side scatter (FSC-A/FSC-H). Cell cycle distribution was determined for wild-type asynchronous cultures and settings were extended to other samples.

☒ Tick this box to confirm that a figure exemplifying the gating strategy is provided in the Supplementary Information.
