# Supplementary material for: TDP1 suppresses chromosomal translocations and cell death induced by abortive TOP1 activity during gene transcription
Source: Nat Commun. 2023 Nov 9;14:6940. doi: 10.1038/s41467-023-42622-7 (PMC10636166; doi:10.1038/s41467-023-42622-7)

Uncropped blots

Figure 1C

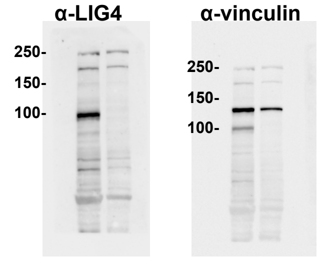

Figure 2A

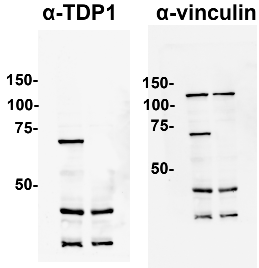

Figure 2H

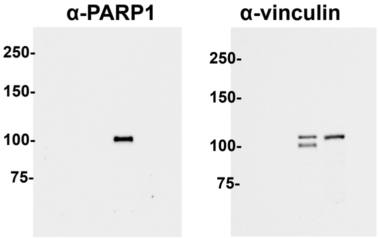

Figures 3A, 3B & 3C

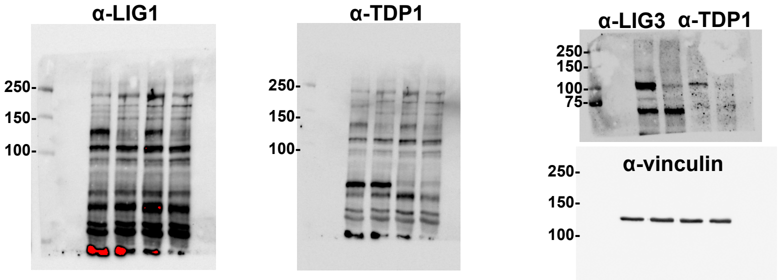

Figure 3D

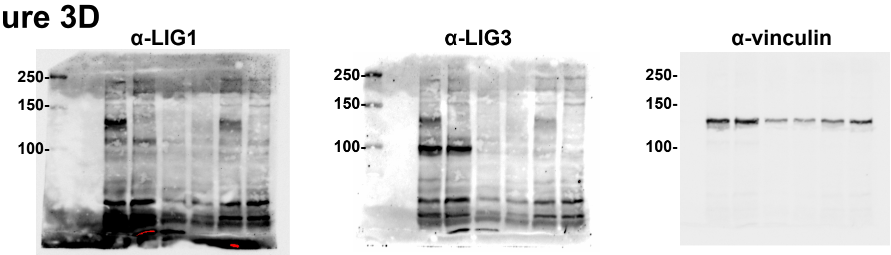

Figure 3E

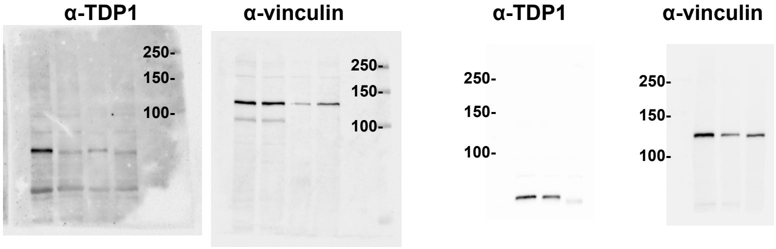

Figure 5B

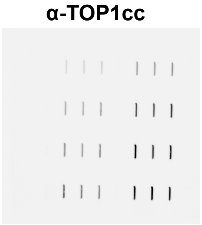

Figure S4

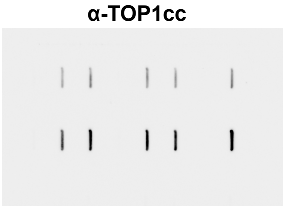

Figure S5A

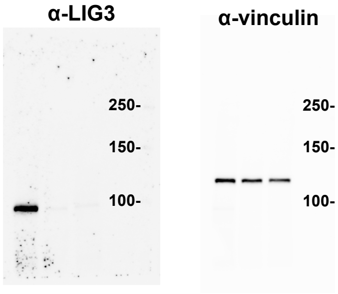

Figure S5B

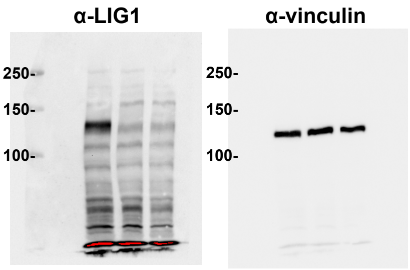

Figure S7A

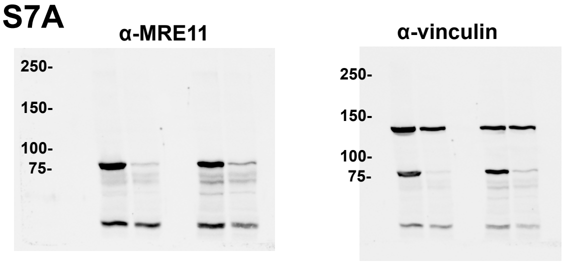

Supplement: Supplementary file 4 — Source Data [file 41467_2023_42622_MOESM4_ESM.zip › Source Data File/Uncropped blots.pdf]
